# Capabilities and Limitations of Fire-Shaping to Produce Glass Nozzles

**DOI:** 10.3390/ma13235477

**Published:** 2020-12-01

**Authors:** Alejandro Rubio, Sergio Rodríguez, Maria G. Cabezas

**Affiliations:** 1 Department of Mechanics, Energetic and Materials Engineering, University of Extremadura, E-06006 Badajoz, Spain; arubiorg@unex.es (A.R.); srodriguwk@alumnos.unex.es (S.R.); 2 Institute of Advanced Scientific Computation (ICCAEx), University of Extremadura, E-06006 Badajoz, Spain

**Keywords:** fire-shaping, fire polishing, glass nozzles, Gas Dynamic Virtual Nozzle, flow focusing, microfluidics

## Abstract

Microfluidic devices for drop and emulsion production are often built using fire-shaped (or fire-polished) glass nozzles. These are usually fabricated manually with inexpensive equipment. The shape limitations and poor reproducibility are pointed as the main drawbacks. Here, we evaluate the capabilities of a new fire-shaping approach which fabricates the nozzle by heating a vertical rotating capillary at the Bottom of a Lateral Flame (BLF). We analyze the effect of the heating conditions, and the capillary size and tolerances. The shape reproducibility is excellent for nozzles of the same size produced with the same conditions. However, the size reproducibility is limited and does not seem to be significantly affected by the heating conditions. Specifically, the minimum neck diameter standard deviation is 3%. Different shapes can be obtained by changing the heating position or the capillary dimensions, though, for a given diameter reduction, there is a minimum nozzle length due to the overturning of the surface. The use of thinner (wall or inner diameter) capillaries allows producing much shorter nozzles but hinders the size reproducibility. Finally, we showed an example of how the performance of a microfluidic device is affected by the nozzle shape: a Gas Dynamic Virtual Nozzle (GDVN) built with a higher convergent rate nozzle works over a wider parametric range without whipping.

## 1. Introduction

Microfluidics is receiving growing attention in numerous fields, especially in biotechnology where it is becoming a key tool. Research in microfluidics involves both the understanding of the physics of flows at such small scales and the development of the devices for different applications. Two-dimensional systems, usually referred to as lab-on-a-chip, have been fabricated by micromachining or soft-lithography. Low cost-high sensitivity diagnosis devices or organ-on-a-chip systems for drug development and testing are some examples [1]. Three-dimensional devices can be built by the assembly of glass capillaries [2,3,4]. The advantages of glass are that it is chemically robust, and has good mechanical and optical properties. It allows building very versatile devices, although their fabrication has a few drawbacks. There are limitations to the nozzle shape, and the assembly and proper alignment of the components may be challenging. Different 3D printing techniques have been also proposed for fabricating microfluidic devices, most with millimetric scale features. However, the resolution improvement of some techniques is broadening their applicability for building real microfluidic devices [5].

Capillary devices in which two nozzle tips are placed in front of each other are very common for emulsion production and encapsulation in the food and pharmaceutical industries [3,4,6,7]. A square shape capillary with an inner diameter equal to the outer of circular ones is used for the alignment. The nozzles are shaped at one end of the round capillaries, and then they are inserted in the square one through opposite ends [6,7,8,9]. To build a fixed microfluidic device, the square shape capillary may be glued onto a microscope slide using an epoxy resin [9]. If disassembly is necessary, standard commercial connectors [8] or dedicated CNC milled blocks with press-fit features [10] can be used to place the capillary.

The Gas Dynamic Virtual Nozzle (GDVN) [11] is another microfluidic capillary-based device which is very commonly used for liquid sample delivery in Analytical Chemistry. It was proposed as an alternative to the original plate-orifice configuration for axisymmetric Flow Focusing (FF) [12]. The GDVN ejector consists of a tapered capillary located inside of a nozzle. Under adequate working conditions, a meniscus of the inner liquid is attached to the capillary tip, and the coflowing stream stretches it dragging a jet much thinner than the nozzle orifice. Assembly and alignment of the components of the GDVN is also an issue. In the original GDVN device, the tube and the capillary were aligned using a centering sleeve [11], which can be replaced by other elements, such as a 3D printed part [13]. The combination of square and round capillaries has also been considered. The outer nozzle was fire-shaped at a square capillary, and the internal needle had an outer diameter equal to the inner square side [14]. In some newer GDVN designs, the outer glass nozzle has been replaced by an injection-molded opaque ceramic one with alignment features to overcome this problem [15,16]. The use of 3D-printing has also been proposed for the direct fabrication of the whole GDVN device. Among all the numerous methods, the 2-photon-polymerization process is preferred due to its high-resolution. The 3D printed GDVNs designs have evolved from the first attempts, which mimicked the original assembly [17], to compact optimized designs with plugging solutions that can be produced within minutes [18].

In capillary-based microfluidic devices, nozzles are usually fabricated by two procedures: pulling or fire-shaping (also known as fire-polishing). The former has been traditionally used for pipette manufacturing. The technology is mature and several commercial pullers are available, which are supplied with a cookbook indicating the adequate process parameter for a particular pipette application or geometry. In pipette pullers, the capillary ends are held by two jaws, and its central section is heated with an electrical resistance or a laser. Then, one or both jaws are moved apart stretching the capillary in the middle and producing two long and needle-shaped pipettes at the same time. To separate them, pulling may be maintained until breakage or a secondary cutting stage may be necessary. Pulled nozzles are commonly used in emulsion devices [9,10]. To obtain adequate geometries, post-processing is usually necessary [19]. The capillaries are pulled to produce a diameter much smaller than the desired one, and then the tips are grazed against abrasive paper to adjust the diameter and significantly reduce the nozzle length. For example, the nozzles used in a gold nanoparticle fabrication device have inner diameters ranging from 100 to 240 μm and are obtained by grazing previously pulled nozzles of 20 μm (the original capillary is 1.0 mm O.D. and 0.58 mm I.D.) [9]. Additionally, as the outer diameter of the nozzle tip also becomes very small, a chemical coating treatment is usually necessary to prevent wetting along the outer surface of the nozzle that would cause malfunctioning of the microfluidic device [9,10].

Fire-shaping produces the nozzle by heating the capillary tip typically with a flame. It is also known as fire-polishing because it is used to smooth the surface of the pipette’s tip after breaking or grazing without significant change in the diameter too. When the capillary tip is heated, the glass flows to reduce its free surface driven by surface tension forces and overcoming the viscous stresses. The material moves inwards reducing the diameter and the length of the capillary. The result is a convergent-divergent nozzle much shorter than a pulled one. The use of these nozzles have extended since Switzer [20] included one in a drop-on-demand device. They have been combined with pulled ones to assemble devices for generating emulsions. The divergent region of fire-shaped nozzles makes them advantageous as collection tubes for correcting misalignment problems [6,21]. When used for injection, they do not need a chemical treatment as their shape creates a favorable contact angle to prevent wetting [8]. To the best of our knowledge, glass nozzles in GDVNs devices are only fabricated by fire-shaping as in the original design [11]. The latter is widely used to produce the jet that carries the samples for femtosecond X-ray protein nanocrystallography [22]. The nozzle was fire shaped in a square shape capillary for the injector for x-ray scattering studies of biological nanospecies [14]. Liquid-liquid flow-focusing with GDVNs [23] can also be used to produce emulsions. In some devices, as in the assembly to produce nanometric polymeric fiber proposed by Ponce-Torres et al. [24], the divergent region of the fire-shaped nozzle is removed.

**Figure 1:**
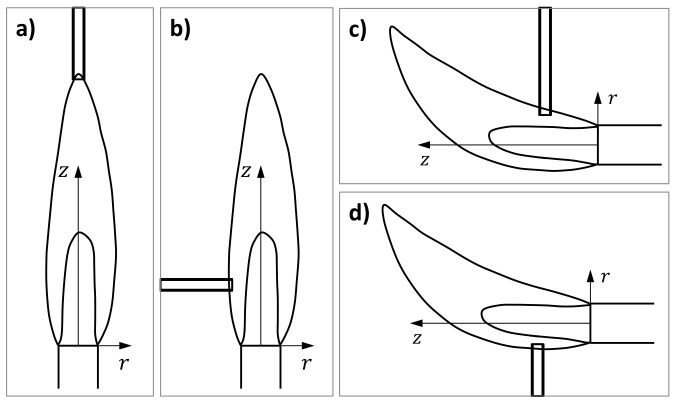
Fire shaping approaches depending on where the capillary is heated: (**a**) Top of a Vertical Flame (TVF), (**b**) Side of a Vertical Flame (SVF), (**c**) Top of a Lateral Flame (TLF) [26], and (**d**) Bottom of a Lateral Flame (BLF). The latter is the approach used in this work.

Although the use of fire-shaped nozzles is extended, most researchers fabricate them manually. Very few fire-shaping systematic studies have been carried out. First, traditional fire-shaping approaches were analyzed using the flame of a vertical Bunsen burner in a setup designed to reduce the operator intervention [25]. When the vertical capillary is heated at the top of the flame (Figure 1a), highly axisymmetric nozzles are produced. However, it does not seem possible to control the nozzle neck diameter with the heating time. Although the neck reproducibility highly increases when the capillary is heated horizontally on the side of the flame (Figure 1b), the lack of symmetry of the nozzles becomes unacceptable. Those results motivated the development of a new approach in which the capillary is heated at the Top of a Lateral Flame (TLF), i.e., the flame produced by a Bunsen burner with its tube horizontal (Figure 1c) [26]. In this way, highly axisymmetric nozzles were produced with satisfactory neck reproducibility. The shape reproducibility at the same position was excellent, and it was possible to obtain different nozzle shapes working at different heating positions.

In this work, we analyze the reproducibility and shape capabilities of a new-fire shaping approach, in which a vertical rotating capillary is heated at the Bottom of a Lateral Flame (BLF) (Figure 1d). The use of this new approach was motivated by the results of preliminary experiments, which showed that the shape variation for different positions was larger than for TLF. We study how the heating position or the dimensions of the original capillary and their variability affect the neck size scattering. The effect of these aspects on the nozzle shape is also explored and the technical limitations are established. BLF is capable of fabricating nozzles with a wide range of shapes. Finally, we show how the nozzle shape may affect the performance of a microfluidic system. In particular, we fabricated two nozzles with significant different shapes and assembled them in a GDVN device for gaseous Flow Focusing. The use of shorter nozzles with a higher convergent rate prevents whipping and extends the pressure range for which it is possible to emit a thin jet.

## 2. Results

Though the fire-shaping idea is simple, the phenomena involved in the process are not. The glass flow that gives rise to the nozzle occurs as the surface tension forces reduce the glass free surface while the viscous forces oppose the flow. The glass viscosity depends highly upon the temperature. The glass cannot stand stress and creeps above the strain temperature (515 ${}^{\circ }$C). The viscosity falls to 10${}^{12}$ Pa·s at 560 ${}^{\circ }$C (annealing point), and to $10^{6.6}$ Pa·s at 825 ${}^{\circ }$C (softening point). Therefore, the viscous resistance to the flow decreases as the temperature rises, larger velocity gradients are allowed in the glass, and the flow becomes faster. On the other hand, we are heating the tip of the capillary at the flame. Heat is transferred from the flame to the glass. The glass temperature rise depends mostly on the amount of glass (capillary thickness) and on the heating time. Conduction losses along the glass are low (the glass thermal conductivity is below 2 W/m K within the temperature range in the experiments). The temperature is maximum at the capillary tip and then decreases along the capillary, and the heating time increases the temperature. When a section is heated up enough, its viscosity lowers enough and it flows. As heating is maintained, the velocity of the flow increases, and the region of the capillary tip heated enough to flow spreads. The velocity of the flow along the capillary, such as the temperature, is not uniform, creating an additional restriction to the flow. Additionally, as the glass flows inwards and downwards, it moves away to a cooler region of the flame, and so the heat transfer is reduced.

### 2.1. Effect of the Heating Time

**Figure 2:**
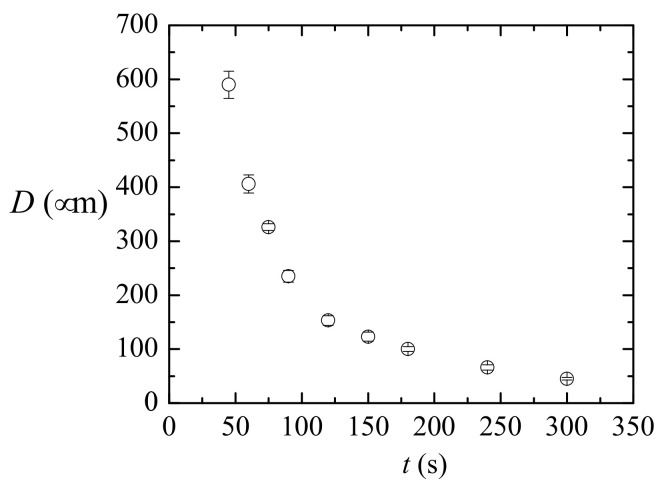
Mean neck diameter for sets of nozzles manufactured at the same heating position (r=3.5 mm, z=15 mm) from capillaries type 1. The standard deviation is used for the error bars.

**Figure 3:**
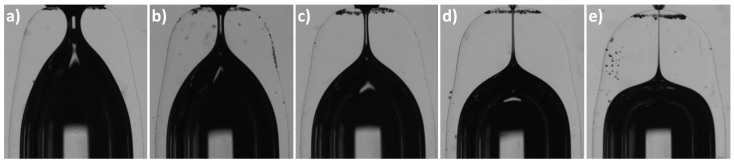
Nozzles manufactured at (r=3.5 mm, z=15 mm) from capillaries type 1: (**a**) t=75 s, D=326 ± 4 μm; (**b**) t=120 s, D=156 ± 1 μm; (**c**) t=180 s, D=102 ± 3 μm; (**d**) t=300 s, D=45 ± 2 μm; (**e**) t=600 s, D<10 μm (not measurable due to limitations of the characterization setup).

**Figure 4:**
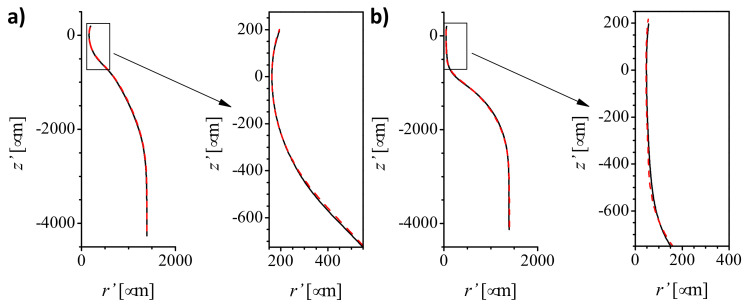
Shape comparison for sets of two nozzles (black and red lines) of the same diameter produced with the same heating conditions from capillaries type 1: (**a**) r=3.5 mm, t=75 s, D≈300 μm; (**b**) r=3.5 mm, t=180 s, D≈100 μm.

Nozzles manufactured under the same heating conditions have scattered neck diameters. This may be caused either by the heating process or by the geometrical tolerances of the capillaries. The importance of these aspects may also depend on the heating conditions (position and time). In the first run of experiments, we analyzed the effect of increasing the heating time for a fixed heating position (r=15 mm). Table 1 and Figure 2 show the mean geometrical parameter for sets of capillaries type 1 heated at the same position (r=3.5 mm, z=15 mm) for different times. The images of some resulting nozzles are shown in Figure 3. As expected, increasing the heating time involves more material in the flow and allows it to flow further. The glass flows inwards reducing the neck diameter *D* and downwards shortening the capillary (increasing ΔL). As it accumulates around the nozzle neck, the neck channel becomes longer (as shown by the increasing neck aspect ratio AR). We did not observe neck channel closure and obtained nozzles with neck diameters of a few micrometers. This is the expected behavior for interfaces when the outer fluid (glass in our case) is very viscous, and the inner fluid (air) has low but finite viscosity [27]. As for the TLF approach [26], the shape reproducibility is excellent. The profiles of nozzles of the same diameter produced with identical heating conditions are indistinguishable (Figure 4). Finally, results show that the heating time does not seem to significantly affect the relative variability of the neck diameter ($s_{D}/\bar{D}$).

### 2.2. Effect of the Geometrical Tolerances of the Capillary

**Figure 5:**
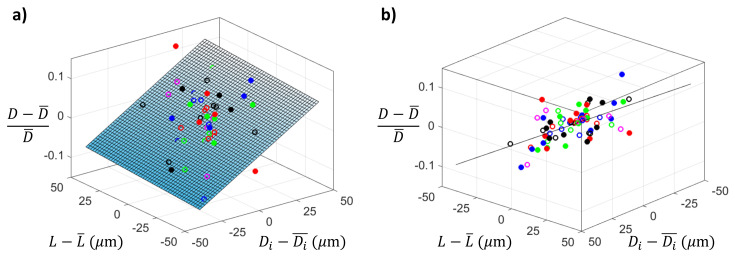
Relative diameter deviation of each nozzle from the mean of the set versus the absolute length and inner diameter deviation of the corresponding original capillary. Symbols correspond to different heating times of the experiments in Table 1 (Capillaries type 1, r=3.5 mm, z=15 mm). The plane is the best fit. Graph (**a**,**b**) represent different view directions.

The neck diameter scattering for the same heating conditions may be caused by (i) the particular dimensions of the original capillary, which vary within the tolerances; or (ii) the variability of the shaping process itself. Figure 5 shows the effect of the actual capillary inner diameter and length on the nozzle neck diameter. For each nozzle of the previous experiment, we calculated the relative deviation of its neck diameter from the mean for its set (heating time), and the corresponding absolute deviation of the capillary inner diameter and length. Symbols correspond to different heating times, ranging from 45 to 300 s. The plane is the fit to the experimental data and shows that the variability of the capillary inner diameter affects the neck diameter more than that of the length. Figure 5b shows a view perpendicular to the plane. The scatter of the values around that plane is still significant, showing that the variation of the capillary dimensions is not the only important source of neck size variability. Uncontrollable changes in the flame heating process affect the final neck diameter.

### 2.3. Effect of the Heating Position

It is possible to produce nozzles of the same neck diameter with different heating conditions. When the nozzle is produced deeper in the flame, a longer region of the capillary is heated up to a higher temperature. Therefore, a larger length of the capillary participates in the flow, and it moves faster. The diameter reduction is reached sooner because of the faster flow, but also because a longer region of the inner surface moves inwards. This effect produces longer nozzles and neck channels. Additionally, as the glass inwards, it also flows downwards producing a larger shortening of the capillary.

**Figure 6:**
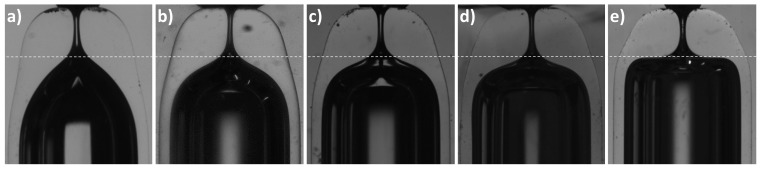
Nozzles of D≈135 μm manufactured at different positions: (**a**) r=3.5 mm, t=150 s, ΔL=1.47 mm; (**b**) r=4 mm, t=250 s, ΔL=1.40 mm; (**c**) r=4.5 mm, t=325 s, ΔL=1.26 mm; (**d**) r=5 mm, t=600 s, ΔL=1.22 mm; (**e**) r=5.5 mm, t=2000 s, ΔL=1.14 mm. The line is to guide the eye.

**Figure 7:**
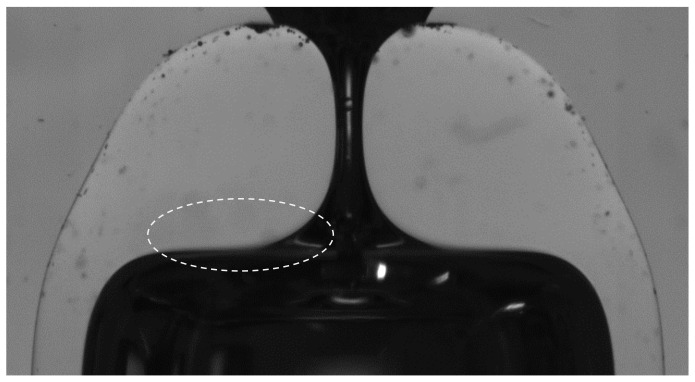
Detail of nozzle presenting overturning (Figure 6e). The slope of the interface shows that the material has flown below the shoulders. The proper inner surface of the nozzle cannot be observed in the image and results in a blur.

**Figure 8:**
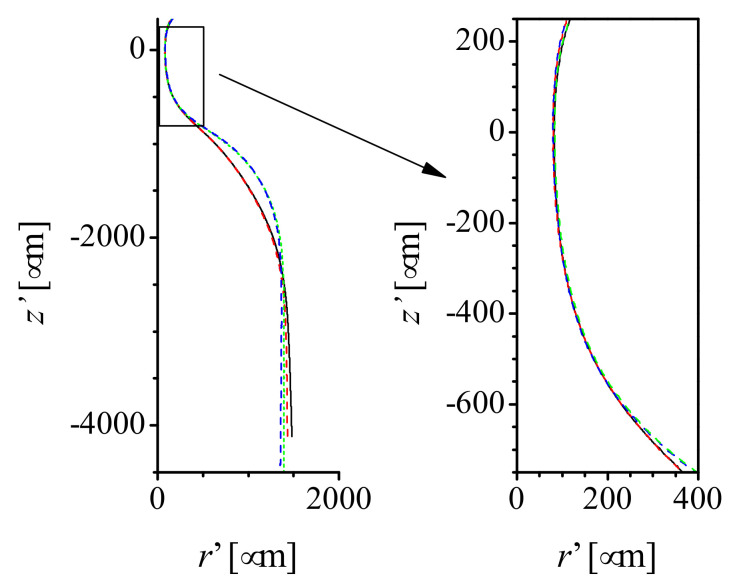
Shape comparison for sets of nozzles with D≈160 μm produced with different heating conditions from capillaries type 1. The heating conditions were (r=3.5 mm, t=120 s) for the black and the red nozzles, and (r=4 mm, t=180 s) for the blue and the green ones.

Table 2 shows the mean geometrical parameters for sets of nozzles of D≃ 135 μm produced at different positions using capillaries type 1. The neck diameter variability for the different heating conditions seems to remain within the same range as that for experiments in Table 1. Therefore, the heating position does not seem to affect significantly the neck diameter reproducibility. However, as expected, it has an important effect on the nozzle shape (see Figure 6). As you work outer in the flame, a shorter region of the capillary is heated up and in a cooler region. The necessary heating time *t* is larger. The glass takes longer to increase the temperature, and the temperature will anyway be lower. The flow will be slower, as shown by the shortening reduction ($\bar{\Delta L}$ decreases about 0.1 mm when you shift the heating position 0.5 mm outer). The capillary length that participates in the flow is smaller. Therefore, less material accumulates around the neck resulting in shorter neck channels (lower $\bar{AR}$). Also, as the diameter reduction happens in a shorter region, the “shoulders” of the nozzle rise. Figure 6e shows what we have called “overturning”. At that position, the length of the capillary heated enough to flow is very short. As the diameter reduction happens as the material moves inwards and downwards, when it is heated long enough, it flows below the nozzle shoulders. This overturning of the interface produces a blur in the image (see Figure 7). Such a shape is not usually adequate for applications. Therefore, in fact, it establishes a limitation of the process: there is a minimum nozzle length that can be obtained for a particular diameter reduction with an appropriate nozzle shape. Again, the shape reproducibility was excellent for nozzles of the same diameter produced in the same conditions (Figure 8).

### 2.4. Effect of the Capillary Geometry: Inner Diameter and Thickness

The capillary geometry affects the shaping process in two ways. First, it affects the heat transfer and the glass temperature for a particular heating position. The outer diameter defines the surface in contact with the flame, and an increase would benefit the heat transfer. On the other hand, the glass temperature will depend on the capillary thickness. For thin capillaries, the glass reaches a higher temperature and flows faster. Second, the geometry of the capillary affects the flow. The heating time is determined by the reduction to be obtained which depends on the inner diameter. Additionally, the capillary outer and inner diameters create restrictions to the flow of the glass free surface.

**Figure 9:**
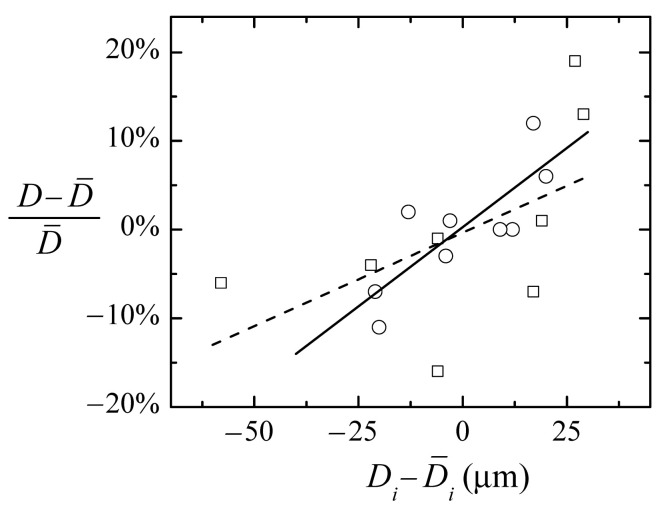
Variability of the neck diameter versus the original capillary inner diameter deviation for thin (circles), and thick (squares) wall capillaries. The solid (dashed) line is the linear fit for the thin (thick) capillaries.

We carried out experiments using different size capillaries to study how the original shape affects the nozzle geometry and the reproducibility of the process. First, we compared capillaries with the same inner diameter and different wall thickness. Table 3 shows the heating conditions and geometrical parameters of sets of nozzles of D≃ 120 μm produced from capillaries type 1 and 2. The thickness of the second almost doubles that of type 1 (see Section 4.2). The cross-section and therefore the amount of material to be heated are larger. It becomes necessary to work deeper in the flame to heat up the glass enough to flow. Still, the glass temperature is relatively low, and the diameter reduction will need a larger heating time. At the heating conditions analyzed (z=15 mm, r=3.5 mm, t=520 s), we need over three times longer to obtain about the same neck diameter with the thicker capillary. On the other hand, one would expect that the material increase would raise the inertia of the process, and compensate for its variability. However, in our experiments, the neck diameter scatter is larger for the thicker capillaries. Figure 9 shows the relative diameter deviation of each nozzle from the mean value of the set versus the absolute inner diameter deviation of the corresponding original capillary for the thin (circles) and the thick (squares) type. As expected, the slope of the trend line is smaller for the thick capillary (dashed line) than for the thin one (solid line). The larger neck diameter scatter has been probably caused by the larger variability in the original dimensions of the capillaries: the measured inner diameter (wall thickness) standard deviation for type 2 was about twice (three times) that corresponding to type 1.

**Figure 10:**
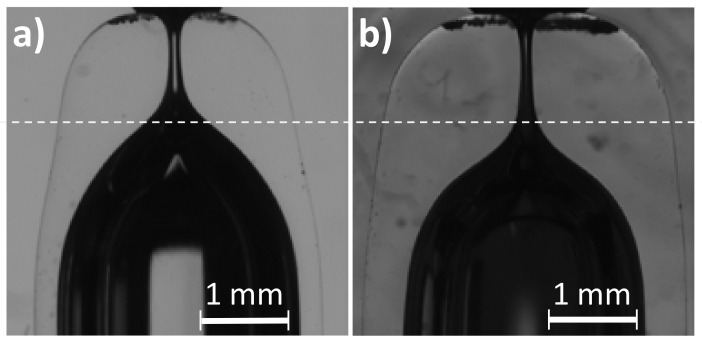
Nozzles of D≈130 μm manufactured from capillaries of the same inner diameter and different wall thicknesses. (**a**) Capillary type 1, r=3.5 mm, t=150 s, ΔL=1.47 mm. (**b**) Capillary type 2, r=3.5 mm, t=520 s, ΔL=1.13 mm. The line is to guide the eye.

The wall thickness also affects the shape of the resulting nozzle. Figure 10 shows two nozzles of the same diameter produced at the same position from a thin (type 1) and a thick (type 2) capillary. As we already mentioned, increasing the thickness slows down the flow and the shortening of the capillary is smaller. However, as the cross-section is greater, the amount of material involved in the flow is still larger, resulting in longer nozzle necks (larger $\bar{AR}$ in Table 3).

Table 4 shows the results of fire shaping smaller capillaries. We used thick (type 3) and thin (type 4) capillaries with a 2 mm outer diameter (see Section 4.2). As their cross-section and/or inner diameter are smaller than for capillaries type 1, the process is very fast, and an outer heating position has to be chosen to be able to control the process (all heating positions in Table 2 are inner than those in Table 4). Type 3 has almost the same cross-section as type 1 but a significantly smaller inner diameter. As the flow has to produce a lower diameter reduction, less material is involved (note that $\bar{\Delta L}$ is much smaller than for all experiments with capillaries type 1). Type 4 has a smaller cross-section than type 3, so with the same heating conditions, the material is heated at a higher temperature. This causes it to flows farther, producing a smaller diameter and a larger shortening, even when the inner capillary diameter is larger. The neck size reproducibility of these two types is quite similar. We checked that the relative variability of the original capillary dimensions stayed within the same range as that for type 1. The causes of the larger neck diameter scattering may be that we work with less material (less inertia), and in a more unstable region of the flame. At the outer heating position studied (r=6.6 mm, z=15 mm) the scatter of the neck diameter becomes unacceptably large.

**Figure 11:**
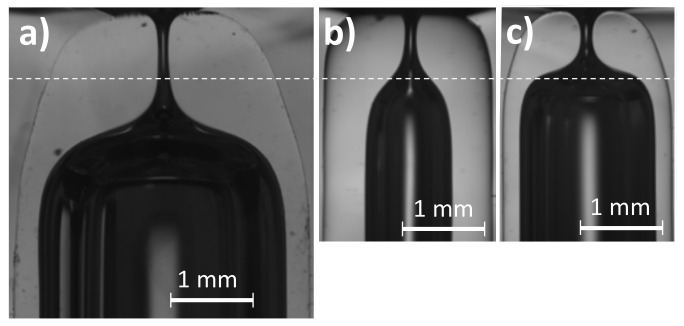
Nozzles of D≈135 μm manufactured from different size capillaries. (**a**) Capillary type 1, r=5 mm, t=600 s, ΔL=1.22 mm; (**b**) Capillary type 3, r=6.6 mm, t=480 s, ΔL=0.11 mm; (**c**) Capillary type 4, r=6.6 mm, t=480 s, ΔL=0.54 mm. The line is to guide the eye.

We tested that the shape reproducibility is maintained for these nozzles. Figure 11 shows nozzles of the same diameter produced in capillaries types 1, 3, and 4. The diameter reduction is much smaller for the latter two, and so is the material involved in the flow. The nozzles are significantly shorter for the smaller capillaries. Please note that, again, the thick-wall capillary (b) produces longer neck channels than the thin one (c), even when the diameter reduction is smaller.

## 3. Discussion

Fire-shaped nozzles are commonly used in microfluidic device assemblies. However, their fabrication is mostly done manually making it difficult to obtain reproducible nozzles or to control its shape. Recently, Munoz-Sánchez and Cabezas [25] proposed an experimental setup to reduce operator intervention in the process and analyzed the traditional vertical flame fire-shaping approaches. Using a non-traditional approach in which the capillary is heated at the top of a lateral flame allowed to improve the nozzle size reproducibility and its axisymmetry [26]. In particular, it was shown that the shape reproducibility was very good, for nozzles of the same diameter produced under the same conditions. In this work, we propose a new approach that heats the capillary at the bottom of the lateral flame (BLF), which additionally, widens the range of nozzle shapes that can be obtained.

The capabilities and limitations of BLF have been experimentally analyzed. First, we focused on neck size reproducibility. For the same heating conditions, the complexity of the flame and glass flow phenomena are only one of the causes of the neck scattering. The variability of the original capillary dimensions, especially the inner diameter, reduces the reproducibility. On the other hand, the heating position does not seem to play a relevant role within a wide region. Unexpectedly, the use of thick wall capillaries did not benefit the size reproducibility in our experiments, probably because the variability in their original dimensions was significantly larger. Reducing the size of the capillaries makes it more difficult to control the process, and results in a larger neck scattering.

We succeeded in producing nozzles of significantly different shapes. Several aspects affect the geometry of the nozzle. As part of an assembly, some of its dimensions may have some limitations. For example, there may be a minimum limit for its inner diameter if there is another element inside of it. If the nozzle has to fit inside another object, there would be a maximum limit for the outer diameter. The thickness may also be relevant as the nozzle has to resist manipulation, assembly, and functioning. The latter may be significant in some applications, for example, those dealing with high viscosity liquids flowing in narrow channels. The neck size is typically determined by the device application and is related to the jet, drop, particle, or bubble size to be produced. Additionally, with the previous parameters fixed, the shape of the nozzle may affect the performance of the device in some applications, as we will see later.

The design reduction (from the inner capillary diameter to the neck) establishes a limit for the nozzle neck shape. It may be smooth, spreading over the nozzle length, or abrupt, converting the nozzle almost into two tubes in series. We defined the neck aspect ratio (AR) to evaluate the length of the neck channel (with diameter below 110% that of the neck) in terms of its diameter. Nozzles for large diameter reductions will show higher AR, though the nozzle itself may be shorter (as the heated material flows inwards and downwards). Both the nozzle and neck channel lengths can be reduced by heating the capillary outer in the flame for a longer time. However, there is a limit for an acceptable shape and a particular reduction. When the heating time is long enough to allow the glass to flow below the heated length, it produces an undesired overturning of the interface. For the same inner diameter, working with thicker capillaries will result in longer channels. Besides, it may limit the shape possibilities, as the heating region for a reasonable heating time narrows. In general, reducing the capillary inner diameter would reduce the diameter reduction and the neck channel length. However, this effect may be compensated for by the wall thickness. We have shown that, for the same outer diameter, thicker capillaries may produce longer channels, even when they need a smaller diameter reduction. With this in mind, the original capillary selection becomes a key aspect determining the nozzle shape and affecting the fabrication reproducibility.

### Application in Flow Focusing to Prevent Whipping

Flow Focusing is a technique that produces very thin jets by hydrodynamic means. The original plate-orifice design [12] to produce axisymmetric flow focusing, has been replaced for several applications by the GDVN ejector. The latter can be built by inserting an inner tube inside of a fire-shaped glass nozzle. The main geometrical parameters are the nozzle neck diameter *D* and its distance to the capillary tip *H*. In gaseous flow focusing, an outer gas stream is used to stretch the meniscus of a liquid formed at the tip of the tube. The air drags the liquid forming a thin jet that travels through the nozzle. That steady jetting is the adequate performance for most applications. Therefore, researchers have studied the parametric region, gas pressure drop Δp and liquid flow rate *Q*, to obtain stable long jets [4]. The jet diameter can be reduced by decreasing *Q* or by increasing Δp. The latter mechanism is limited by whipping instability [28,29,30]. When the energy transferred to the jet is too high, the jet oscillates as a whip. Global whipping is an undesired phenomenon, not present in the original plate-orifice configuration that was observed in GDVN devices and can hinder their applicability. When the pressure drop rises, the meniscus starts oscillating laterally. This may even cause the interruption of the jet if it touches the inner nozzle wall. To prevent this effect, it was suggested to use nozzles with higher convergent rates [28].

**Figure 12:**
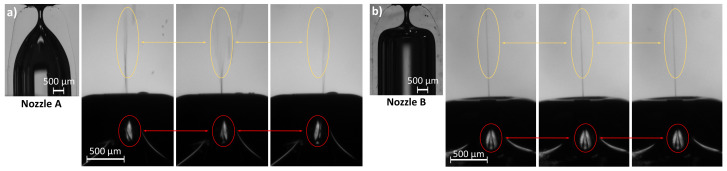
Sequences of experimental images of the jet emitted by the two GDVNs devices with (**a**) Nozzle A and (**b**) Nozzle B. The water inner flow rate is Q=6.9 mL/h and the air pressure drop is Δp=300 mbar.

**Figure 13:**
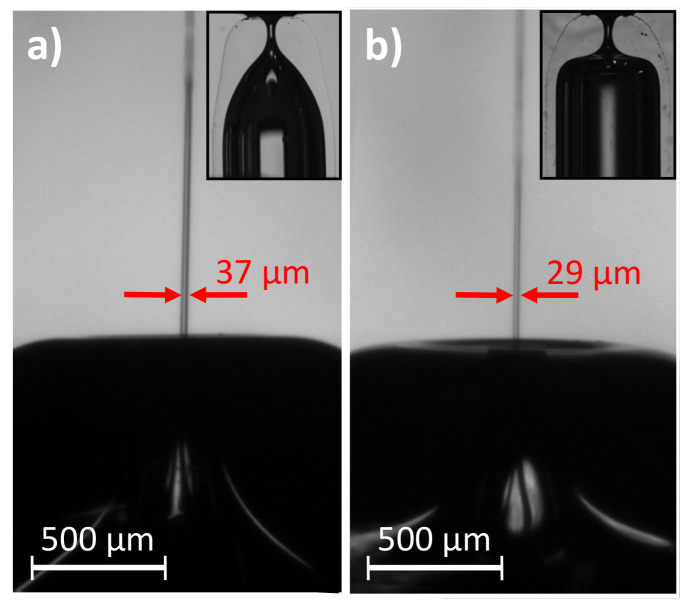
Steady jet emitted by the two GDVNs devices with (**a**) Nozzle A and (**b**) Nozzle B. The water inner flow rate is Q=20 mL/h and the air pressure drop is Δp=150 mbar.

We have fabricated two GDVN devices differing only in the nozzle shape and tested their performance. Distilled water was injected through the inner tube at Q=6.9 mL/h, and the outer stream was produced by a pressure drop Δp=300 mbar. Figure 12 shows the two nozzles and a sequence of images of the jet emission for the same working conditions. For Nozzle A, global whipping is observed. Both the jet, marked with a yellow circle, and the meniscus, in the red circle, oscillate significantly. On the contrary, Nozzle B, which has a more abrupt shape, produces a steady jet. There is a range of experimental conditions for which both GDVNs devices emit a steady jet. However, its size (and velocity) are also affected by the nozzle shape. Figure 13 shows the water jets emitted by both devices at Q=20 mL/h, and with a pressure drop Δp=150 mbar. Nozzle B device produces a 20% thinner jet. A deeper investigation of the effect of the nozzle shape for different liquid-air systems and working conditions can be found in [31].

## 4. Materials and Methods

### 4.1. Fire-Shaping Setup

**Figure 14:**
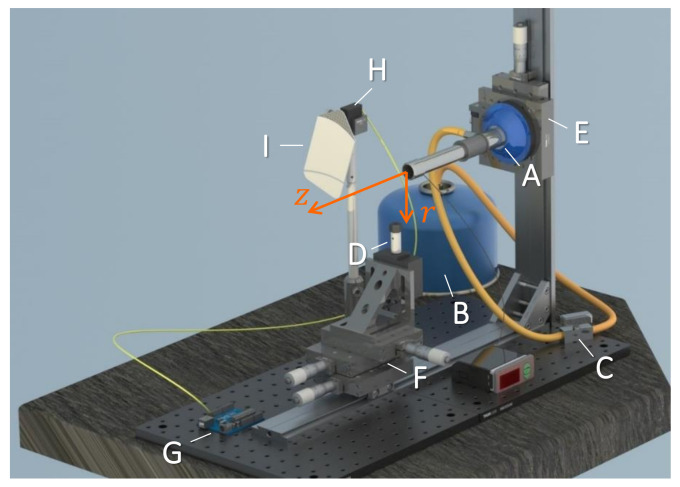
Fire-shaping setup: (A) Bunsen burner, (B) gas bottle, (C) gas valve, (D) collect and chuck holder, (E) vertical translation stage, (F) horizontal two-axis translation stage, (G) Arduino board, (H) servomotor and (I) ceramic plate.

Figure 14 shows the setup used in the experiments. The flame is produced by a Bunsen burner (JP Selecta, Abrera, Spain, ref. 7000134) (A) with its tube placed horizontally. The burner slots are fully open to produce a hotter flame (with maximum air proportion in the mixture). The flammable gas is butane supplied through a hose that connects the burner to a commercial gas bottle (B). A self-locking gas valve (C) installed in the hose is used to allow or shut off the butane flow. A 0.8 kg/h regulator fitted to the gas bottle ensures a constant gas flow in all the experiments. The capillary is held vertically and attached to a DC motor using a suitable collet and chuck holder set (D). The burner is mounted on a translation stage (E) that moves vertically to control the distance *r* of the capillary tip to the burner tube axis. A two-axis translation stage (F) allows us to move the capillary horizontally for proper alignment and to control the distance *z* of the capillary axis to the burner tube exit. The heating time *t* is controlled by an Arduino board (G) and a servomotor (H) which places a ceramic plate (I) between the burner and the capillary or moves it away.

**Figure 15:**
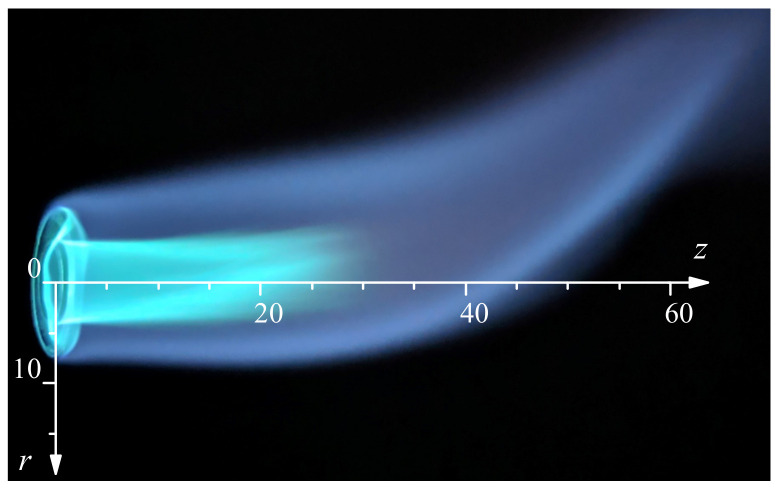
Image of the flame and coordinate system for the heating position.

**Figure 16:**
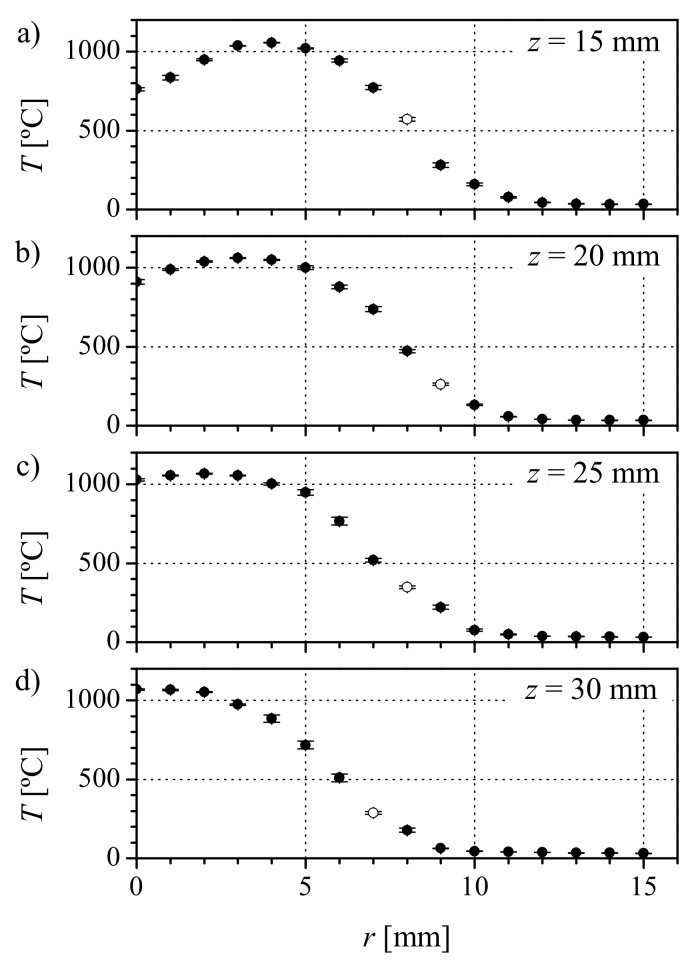
Measured temperature profiles at the flame at different distances to the Burner exit: (**a**) z=15 mm, (**b**) z=20 mm, (**c**) z=25 mm, and (**d**) z=30 mm. The white symbol corresponds to the limit of the visible flame.

Figure 15 shows an image of the lateral flame and the coordinate system used to define the capillary tip heating position. We used a mineral insulated type K thermocouple (1 mm in diameter) to estimate the temperature at different flame positions. The thermocouple tip was moved to a particular position and, after one minute, 100 hundred measurements were taken at one measurement per second. The measured value is not the flame temperature (it underestimates it), nor the glass temperature during the shaping. However, it can be used to estimate the heat capacity of the flame. Figure 16 shows the temperature profiles at different distances *z* from the burner exit. The symbols represent the mean value of the measurements, and the standard deviation is used for the error bars. The white symbols correspond to the visible limit of the flame. The presence of the blue cone, which is a cold area, can be appreciated close to the burner (z<25 mm). The temperature rises from room temperature far away from the flame, reaches a maximum at the blue region border, and then decreases as you move towards the burner axis. The maximum temperature is measured around the tip of the blue cone (r=0 mm, z=30 mm). However, to place the capillary tip at that position would mean to heat a very long region of the capillary. Close to the burner, the temperature increment spreads over a shorter distance. Therefore, that position seems to be more adequate to produce short nozzles and to control the nozzle shape. Preliminary experiments confirmed that, for different radial positions, larger variations in the shape were obtained when working closer (lower *z*) to the burner. Therefore, we chose the closest possible position (z=15 mm) and maintained it for all the experiments.

For each heating conditions (r,t), we manufactured a set of 8 nozzles with the following procedure. First, the heating time is loaded into the Arduino board, and the gas bottle and the burner valves are fully opened. The capillary is placed and tightened with the chuck holder set. Then, the cycle begins when the motor rotation is started and the ceramic plate moves to the isolation position. That allows the operator to open the gas valve and start the flame with a spark lighter before the ceramic plate moves away. The heating starts at that moment and finishes when the ceramic plate is moved back between the capillary and the flame. The operator extinguishes the flame by closing the gas valve. Meanwhile, the capillary remains rotating for the programmed cooling time. Finally, the capillary is released from its holding.

### 4.2. Capillaries and Nozzles Geometries

To manufacture the nozzles we used cut-end glass capillaries made of borosilicate glass 3.3 (Hilgenberg GmbH). The dimensions and tolerances, according to the supplier, are shown in Table 5.

We used image analysis to obtain the shape and several geometrical parameters of the nozzles. We take three images of each nozzle with its tip submerged in glycerine and air flowing through it. The air inside the nozzle appears black in the image, making edge detection easier (Figure 3 images of some nozzles). The glycerine bath is used to reduce optical distortion. Then, two (left and right) nozzle contours are obtained for each image by a several-step procedure. The Canny edge detector is used at the pixel level, and a local intensity threshold and interpolation are used to achieve subpixel resolution. Points that are too far from the original pixel are rejected. Finally, the symmetry axis is determined and a single mean profile is calculated. Figure 4 shows the mean profiles corresponding to two different nozzles manufactured with the same conditions. Also, we obtain the nozzle neck diameter *D*, the outer diameter $D_{o}$ and inner diameter $D_{i}$ of the capillary, as well as the neck length $L_{n}$, defined as the length of the channel with a diameter below 110% the neck diameter. From those parameters, we calculate the capillary thickness $T=D_{o}-D_{i}$, and the neck aspect ratio $AR=L_{n}/D$. Additionally, we calculate the capillary shortening $\Delta L=L-L_{f}$ due to fire shaping from the length of the capillary before *L* and after $L_{f}$ fire shaping measured with a caliper. For each set of nozzles manufactured in the same conditions, we calculated the mean $\bar{P}$ and the standard deviation $s_{P}$ for all the geometrical parameters *P* mentioned above.

### 4.3. Gaseous Flow-Focusing

We built two GDVNs ejectors for gaseous flow-focusing differing only in the outer glass nozzle shape. Both nozzles were fabricated from capillaries type 1 following the procedure described in Section 4.1. Nozzle A was fabricated deep in the flame with a short heating time, while Nozzle B was fabricated outer with a much longer time to obtain the same diameter (Table 6). This way, the diameter transition extends over a long region in the former and is more abrupt in the latter (Figure 12).

**Figure 17:**
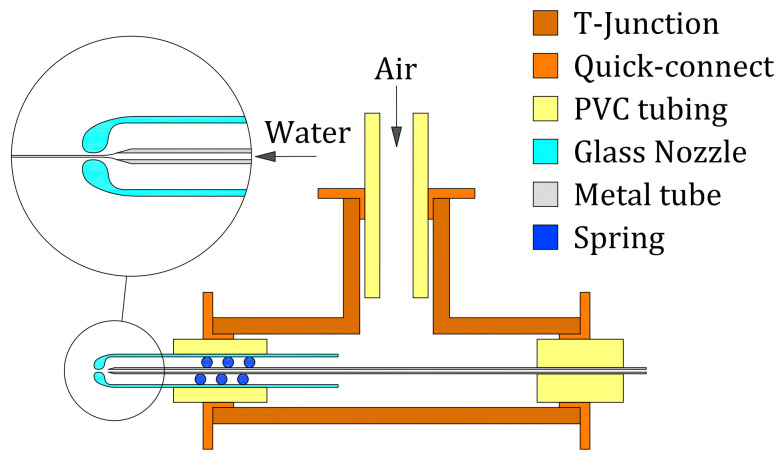
Assembly of the GDVN device.

We used a pneumatic tee connector (SMC, KQ2T06-00A) with instant fittings to assemble the device (Figure 17). The glass nozzle was mounted on one side of the T-junction, and the inner tapered metal tube (400 μm OD, 200 μm ID, and 14${}^{\circ }$ angle) was inserted through the opposite side. Fitting and sealing were achieved by using adequate PVC tubing. The tube was aligned using a spring and its exit was located at a distance H=450 μm to the nozzle neck (D≈220 μm). The lateral port of the T-junction was connected to a compressed air source through a high accuracy pressure regulator valve, which allowed to control the pressure drop Δp of the coflowing air stream through the nozzle. The water was injected through the inner tube at a controlled flow rate *Q* using a syringe pump (KDS120, kdScientific, Holliston, MA, USA). A CCD camera (AVT Stingray F-125B, Allied Vision, Stadtroda, Germany) and backlighting were used to obtain images of the jet.

## Figures and Tables

**Table 1:** Mean geometrical parameters for sets of nozzles manufactured at the same position (r=3.5 mm, z=15 mm) from capillaries type 1.

*t* (s)	$\bar{D}$ (μm)	$s_{D}/\bar{D}$ (%)	$\bar{\Delta L}$ (mm)	$\bar{\mathrm{AR}}$
45	590	4	1.08	0.47
60	406	4	1.21	0.62
75	326	2	1.26	0.78
90	235	4	1.36	1.04
120	153	6	1.48	1.78
150	123	7	1.50	2.55
180	100	4	1.57	3.65
240	66	7	1.65	7.13
300	45	5	1.72	10.1

**Table 2:** Mean geometrical parameters for sets of nozzles of approximately the same diameter produced with different heating conditions. Capillaries type 1, z=15 mm.

*r* (mm)	*t* (s)	$\bar{D}$ (μm)	$s_{D}/\bar{D}$ (%)	$\bar{\Delta L}$ (mm)	$\bar{\mathrm{AR}}$
3.5	150	123	7	1.50	2.55
4.0	250	135	5	1.39	2.46
4.5	325	140	4	1.29	1.81
5.0	600	140	3	1.20	1.73

**Table 3:** Mean geometrical parameters for sets of nozzles fabricated from capillaries of different thicknesses (z=15 mm).

Type	*r* (mm)	*t* (s)	$\bar{D}$ (μm)	$s_{D}/\bar{D}$ (%)	$\bar{\Delta L}$ (mm)	$\bar{\mathrm{AR}}$
1	3.5	150	123	7	1.50	2.5
2	3.5	520	115	12	1.28	3.6

**Table 4:** Mean geometrical parameters for sets of nozzles fabricated from different capillary types and with different heating conditions at (z=15 mm).

Type	*r* (mm)	*t* (s)	$\bar{D}$ (μm)	$s_{D}/\bar{D}$ (%)	$\bar{\Delta L}$ (mm)	AR
3	5.5	70	70	19	0.25	4.2
3	6	70	239	12	0.12	1.0
3	6.6	480	221	36	0.08	1.2
4	5.5	70	55	14	0.77	4.6
4	6	70	95	18	0.65	2.6
4	6.6	480	122	29	0.54	1.8

**Table 5:** Nominal dimensions and tolerances of the capillaries.

Type	$D_{o}$ (mm)	$D_{i}$ (mm)	*T* (mm)	*L* (mm)
1	3.3 ± 0.1	2.773 ± 0.1	0.264	100 ± 0.5
2	3.7 ± 0.1	2.775 ± 0.1	0.462	100 ± 0.5
3	2.0 ± 0.1	1.0 ± 0.1	0.5	100 ± 0.5
4	2.0 ± 0.1	1.6 ± 0.1	0.2	100 ± 0.5

**Table 6:** Heating conditions and geometrical parameters of the nozzles assembled in the GDVNs devices (Figure 12).

Noozle	*r* (mm)	*z* (mm)	*t* (s)	*D* (μm)	AR
A	3.5	15	90	224 ± 2	1.07
B	5	15	700	218 ± 3	0.96
